# An Apparatus to Enable Deaf Persons to Hear Public Speakers

**Published:** 1890-07

**Authors:** 


AN APPARATUS TO ENABLE DEAF PERSONS TO HEAR PUBLIC SPEAKERS.—Dr. Francis H. Brown describes in the *Boston Medical and Surgical Journal*, a device which enables a deaf person to hear the sermon and other services at church. It consists of a trumpet-shaped tin sound-receiver having an opening superiorly of six inches, and narrowing inferiorly to a diameter of one inch. Its upper end is curved, so that the opening of the trumpet is directed toward the mouth of the speaker. The sound-receiver stands on the desk or pulpit, and is partially concealed by a gas-fixture. At the lower end, it fits into the opening of a common tin speaking-tube, which passes down through and under the floor to the seat of the lady in the audience; at that point the tube ends in a piece of brass pipe, rising some four inches above the seat, over which the person using it places the bell-shaped end of the flexible conversation tube now very commonly used by the deaf.—*Memphis Medical Monthly.*

